# Orofacial Neuropathic Pain-Basic Research and Their Clinical Relevancies

**DOI:** 10.3389/fnmol.2021.691396

**Published:** 2021-07-06

**Authors:** Masamichi Shinoda, Yoshiki Imamura, Yoshinori Hayashi, Noboru Noma, Akiko Okada-Ogawa, Suzuro Hitomi, Koichi Iwata

**Affiliations:** ^1^Department of Physiology, Nihon University School of Dentistry, Tokyo, Japan; ^2^Department of Oral Diagnostic Sciences, Nihon University School of Dentistry, Tokyo, Japan

**Keywords:** trigeminal nerve injury, trigeminal ganglion, trigeminal spinal subnucleus caudalis, craniofacial pain, oral diagnosis, treatment

## Abstract

Trigeminal nerve injury is known to cause severe persistent pain in the orofacial region. This pain is difficult to diagnose and treat. Recently, many animal studies have reported that rewiring of the peripheral and central nervous systems, non-neuronal cell activation, and up- and down-regulation of various molecules in non-neuronal cells are involved in the development of this pain following trigeminal nerve injury. However, there are many unknown mechanisms underlying the persistent orofacial pain associated with trigeminal nerve injury. In this review, we address recent animal data regarding the involvement of various molecules in the communication of neuronal and non-neuronal cells and examine the possible involvement of ascending pathways in processing pathological orofacial pain. We also address the clinical observations of persistent orofacial pain associated with trigeminal nerve injury and clinical approaches to their diagnosis and treatment.

## Introduction

Peripheral nerve injury produces high-frequency injury discharges in the injured nerve fibers. These high-frequency injury discharges in the primary afferent neurons are conveyed to the central nervous system via dorsal root ganglion neurons, resulting in the enhancement of nociceptive neurons in the spinal dorsal horn. In the trigeminal system, injury discharges are conveyed to the trigeminal spinal subnucleus caudalis (Vc) and upper cervical spinal cord (C1-C2) via trigeminal ganglion (TG) neurons, resulting in severe persistent pain in the orofacial region (Dubner and Ren, 2004). In association with hyperactivation of TG, Vc and C1-C2 nociceptive neurons, non-neuronal glial cells and macrophages are activated and accumulated, respectively in the TG, Vc, and C1-C2 regions. Neuron-non-neuronal cell communication is thought to be involved in the enhancement of macrophage accumulation and activation of non-neuronal glial cells, and further causes the increase of neuronal cell activation. It is also known that activated satellite cells, microglial cells, and accumulated macrophages generate a variety of cytokines, neurotrophic factors, and tumor necrosis factors in Vc and C1-C2 regions and are released from non-neuronal glial cells and macrophages (Liu et al., 2000; Ristoiu, 2013).

These neuron-non-neuronal cell communications are also considered to be involved in the acceleration of spreading of the neuronal activation in TG, Vc, and C1-C2 regions. Activated satellite glial cells and accumulated macrophages release various cytokines and cause further enhancement of the excitability of uninjured TG neurons, resulting in the spreading of the activation of TG neurons. Further, in Vc and C1-C2 regions, microglia-astrocyte interaction is thought to be involved in the spreading of the excitability of nociceptive neurons in Vc and C1-C2 (Shibuta et al., 2012; Asano et al., 2020).

Noxious information from the brain stem neurons is further conveyed to the higher CNS areas (Iwata et al., 2017b). Three major CNS areas receiving noxious inputs from Vc and C1-C2 regions are known to be involved in processing orofacial pathological pain, ventral posteromedial thalamic nucleus (VPM), medial thalamic nuclei (MT), and parabrachial nucleus (PBN) (Saito et al., 2017). It has recently been shown that these ascending pathways are functionally modulated after trigeminal nerve injury (Okada et al., 2019).

Trigeminal nerve injury is known to causes long-lasting, widely spreading, and persistent pathological pain in orofacial regions in humans. However, we do not have an appropriate strategy to diagnose and treat these orofacial pain because detailed mechanisms underlying pathological pain associated with trigeminal nerve injury are not fully understood. It is essential to know the mechanisms underlying these pain for the development of the appropriate treatment of orofacial neuropathic pain patients.

In this review, we address recent animal data regarding the involvement of neuron-non-neuronal cell communication and related various molecules and also address possible involvement of ascending pathways for processing of the orofacial pathological pain associated with trigeminal nerve injury. We also demonstrate the clinical observations of orofacial neuropathic pain and trigeminal neuralgia patients and their clinical approaches to diagnose and treat these orofacial neuropathic pain patients.

## Peripheral Mechanisms of Orofacial Neuropathic Pain

Trigeminal nerve injury results in pain hypersensitivity categorized as persistent orofacial pain, allodynia, and hyperalgesia (Kaji et al., 2016; Batbold et al., 2017; Sugawara et al., 2019). When the peripheral nerves are damaged, the cellular response is induced by via various molecules at the site of nerve damage. In the first place, nerve inflammatory response is triggered in relation to several changes in the microenvironment of immune cells surrounding the damaged peripheral nerves. Concurrent with immune cells infiltration to the injury site, some inflammatory molecules which cause the hypersensitivity of the peripheral nerve are released from the damaged neurons and Schwann cells. Moreover, changes in neuronal excitability with regard to threshold decrease and spontaneous firing are caused in the damaged neurons (Peng et al., 2016). For example, in greater detail: the levels of proinflammatory mediators like tumor necrosis factor-alpha (TNFα) or nerve growth factor are upregulated at the sites of peripheral nerve injury (Chu et al., 2020). TNFα binds to TNF receptor (TNFR), expressed in non-injured nerve endings, which results in a change in the excitatory potential of voltage-gated sodium channels 1.8 (Nav1.8), via activation of protein kinase C, leading to neuronal hyperexcitation (Leo et al., 2015). Another pathway to hyperexcitation can be found in tongue mucosa collected from patients with burning mouth syndrome, which is thought to have neuropathic involvement at various levels of the neuraxis: the mRNA expression in artemin, which is one of the glial cell line-derived neurotrophic factor, is upregulated (Shinoda et al., 2015). The upregulated artemin signaling leads to the hyperexpression of TRPV1 in tongue nociceptors via the p38 mitogen-activated protein kinase phosphorylation, producing heat hypersensitivity in the tongue (Shinoda et al., 2015). Many monocyte-derived macrophages are known to infiltrate at the nerve injury site following peripheral nerve injury. These blood-borne macrophages accumulate especially around injured axons, the accumulation is caused by monocyte chemoattractant protein-1 (MCP-1) signaling, that modulates the development of neuropathy (Kanamori et al., 2007). Reportedly, peripheral nerve injury induces infiltration and proliferation of macrophages which release insulin-like growth factor-1 (IGF-1) (Liu et al., 2000). Via TRPV2, the signaling of IGF-1 that is released by macrophages which accumulate in the site of infraorbital nerve injury, increases TRPV4 expression in neurons of the TG, innervating the facial skin. This results in mechanical hypersensitivity in the facial skin (Sugawara et al., 2019). Many molecules released from the injured axons, the infiltrating macrophages, and Schwann cells activate non-injured axons after peripheral nerve injury. The activation of non-injured axons might play an important role in peripheral neuronal hyperexcitability, closely correlated with neuropathic pain.

## Transcellular Communication Between Neurons and Inflammatory Cells in TG

Satellite glial cells, lymphocytes, macrophages, and the somata of the primary afferent neurons, are known to be present in TG. The satellite glial cells surround the sensory neuronal soma, and these cells communicate with each other via neurotransmitters. Nerve injury is reported to activate cell-to-cell communication in TG, via humoral factors, including cytokines, neuropeptides, and gas. Interestingly, macrophages accumulate at the site of peripheral nerve injury and in TG, where the somata of the injured primary afferent neurons are found (Shinoda et al., 2019). Additionally, trigeminal nerve injury leads to infiltration of inflammatory cells, including macrophages, into TG and their subsequent accelerated activation. Following peripheral nerve injury in the orofacial region, TNFα or Substance P (SP) is released from the accumulated macrophages in TG, which leads to TG neuronal hyperexcitability followed by orofacial pain hypersensitivity (Batbold et al., 2017). The proliferation of resident macrophages is accelerated in sensory ganglia following peripheral nerve injury (Donegan et al., 2013). Some orofacial pathological conditions induce characteristic morphological changes in the infiltrated and resident macrophages (thicker ramifications and a larger soma), accelerating the release of various neurotransmitters. Thus, it is presumed that changes in the morphological appearance indicate activation of the macrophages.

Additionally, macrophages can be divided into two histological types, depending on their specific functional properties. First, the classically activated phenotype known as the M1 macrophage, which releases a variety of proinflammatory mediators, plays an essential role in the early stages of inflammatory reaction. Second, the alternatively activated phenotype is known as the M2 macrophage which has an anti-inflammatory effect and is associated with the tissue repair process. Peripheral nerve injury elicits infiltration and activation of M1 and M2 macrophages at the site of nerve damage and in the sensory ganglion where the somata of the injured neurons lie (Komori et al., 2011). In TG, transcellular communication between neurons and macrophages, via various biochemical mediators, regulates the excitability of TG neurons following peripheral nerve injury in the orofacial region (Iwata et al., 2017a). Trigeminal nerve injury induces infiltration of macrophages which release TNFα into TG. The TNFα signaling contributes to TG neuronal hypersensitivity, resulting in orofacial neuropathic pain (Batbold et al., 2017). Following peripheral nerve injury, a wide variety of biochemical mediators released from TG neurons also mediate the accumulation and activation of M1 and M2 macrophages. For example, the chemokine C-C motif ligand 2 (CCL2) is released from the somata of injured TG neurons, and the CCL2 signaling activates macrophages that accumulate in TG (Kim et al., 2011; Liu et al., 2016).

## Communication Between Satellite Cells in TG

Recent studies have indicated that peripheral nerve injury leads to functional and morphological changes in satellite cells (swelling of soma and shortening of processes), and these changes confer the primary neuronal hyperactivity (Li and Zhou, 2001). Moreover, satellite cells communicate with each other via gap junctions, which allow various molecules to pass between cells (Hansson and Skioldebrand, 2015).

Connexin 43 (Cx43), a gap junction protein, regulates the transport of several molecules between satellite glial cells (Chen et al., 2012). In TG, morphological changes of satellite glial cells are induced by inferior alveolar nerve injury via Cx43, resulting in extensive orofacial mechanical hypersensitivity (Kaji et al., 2016). These reports indicate that satellite glial cells are activated via Cx43 throughout TG, playing an essential role in ectopic orofacial pain via the enhancement of trigeminal neuronal excitability.

Thus, non-neuronal cell mechanisms in the TG might induce ectopic or extraterritorial pain hypersensitivity associated with peripheral nerve injury in the orofacial region. The orofacial ectopic pain can easily lead to misdiagnosis as dental pain and unnecessary and irreversible dental treatment such as pulpectomy or tooth extraction. Further elucidation of these mechanisms might make management of orofacial neuropathic pain and avoidance of misdiagnosis much easier for the clinician treating orofacial neuropathic pain.

## Brainstem and Cervical Spinal Cord Mechanisms

**FIGURE 1:**
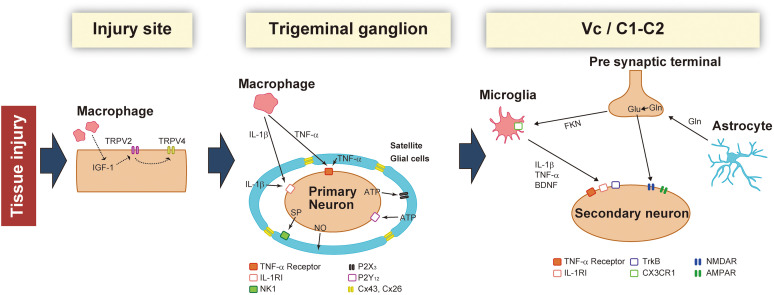
Schematic illustration of the changes that occur in the injured site, trigeminal ganglion (TG), and Vc/C1-C2 following trigeminal nerve injury. After nerve injury, macrophages sensitize TG neurons by activating peripheral nerves and the soma. In the TG, neuronal activity is potentiated by secretory factors from satellite glial cells. Activated glial cells such as microglia and astrocytes further enhance synaptic transmission in the Vc and C1-C2.

**FIGURE 2:**
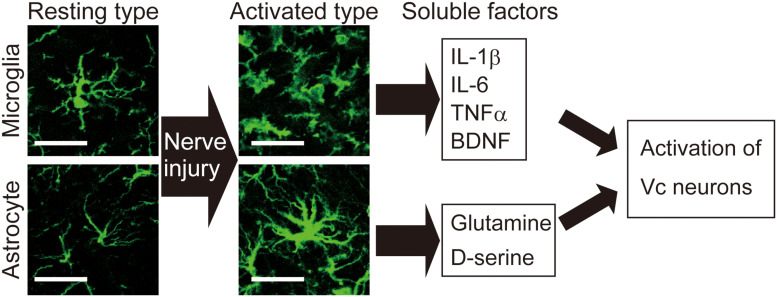
Activation of microglia and astrocytes is observed in the Vc following peripheral nerve injury. Images show microglia and astrocytes in the Vc of normal rat or rat with infraorbital nerve injury (IONI). In the physiological situation, both microglia and astrocytes exhibit highly branched and thin processes, which is called resting type. Seven days after IONI, hypertrophic morphology of microglia and astrocytes, which is called activated type, is observed in the Vc. Activated types of glial cells release multiple factors that enhance neuronal activity in the Vc. Scale bar: 50 μm.

The trigeminal spinal nucleus is an elongated structure that is divided into three subnuclei: oralis, interpolaris, and caudalis. The bulk of the trigeminal spinal nucleus is taken up with Vc, which has a laminated structure similar to that of the spinal cord. Nociceptive information arising from the craniofacial region is conveyed to the Vc, and the C1-C2, via primary afferent fibers, the somata of which lie in TG. Projection neurons, carrying nociceptive information from the craniofacial region, are found in the branches of the trigeminal nerve. The ophthalmic and mandibular branches of the trigeminal nerve project to the ventral and dorsal portion of both C1-C2 and Vc, respectively. The maxillary branch projects between the ventral and dorsal. Noxious information from brainstem neurons is further conveyed to the higher CNS areas (Iwata et al., 2017b). Three major CNS areas receiving noxious inputs from Vc and C1-C2 regions are known to be involved in processing orofacial pathological pain: posterior medial thalamic nucleus, MT, and PBN (Saito et al., 2017). The orofacial noxious pathway projecting to VPM is thought to be involved in the sensory discriminative aspect of pain. In contrast, the MT and PBN pathways are considered to be involved in the motivational and affective aspects of pain (Saito et al., 2017). Recently, these two ascending pathways have been functionally altered after trigeminal nerve injury (Okada et al., 2019). In response to trigeminal nerve injury, increased activity in *N*-methyl-D-aspartate (NMDA) and alpha amino-3-hydroxy-5-methyl-4-isoxazole-propionate (AMPA) receptors were found to facilitate synaptic transmission (Dubner and Ren, 2004). GABAergic and glycinergic inhibitory neurotransmission have also been seen to change following trigeminal nerve injury (Okada-Ogawa et al., 2013). In recent years, accumulated evidence has clarified that glial cells in the central nervous system are essential factors in inducing a wide variety of changes in neuronal function in the Vc following trigeminal nerve injury. Microglia and astrocytes are key players in neuropathic pain and are activated in response to trigeminal nerve injury as well as inflammation in the orofacial region (Okada-Ogawa et al., 2009; Figure 1). It is generally believed that microglia are activated in the early phase of nerve injury and that astrocytic activation occurs subsequently. Microglia are macrophage-like immune cells, secrete several proinflammatory cytokines, including interleukin (IL)-1β, IL-6, and TNFα. These cytokines can enhance both the frequency and amplitude of spontaneous excitatory postsynaptic currents (sEPSCs) in lamina II spinal neurons (Kawasaki et al., 2008). Among these cytokines, the target of IL-1β is the phosphorylation of NMDA receptors (Guo et al., 2007). Proinflammatory cytokines inhibit both GABA- and glycine-mediated spontaneous inhibitory postsynaptic currents (sIPSCs) in lamina II spinal neurons (Kawasaki et al., 2008). Another secretory molecule from microglia is the brain-derived neurotrophic factor (BDNF), which plays an essential role in neuropathic pain. BDNF binds to tropomyosin-related kinase B (TrkB), and in turn, leads to a reduction in expression levels of K^+^-Cl^–^ cotransporter (KCC2). Due to the accumulation of intracellular Cl^–^, GABA receptor-mediated Cl^–^ influx changes to Cl^–^ efflux, resulting in the excitatory response by GABA (Coull et al., 2005). Down-regulation of KCC2 in the Vc is observed after 21 days of inferior alveolar nerve injury in rat model of inferior alveolar transection, which results in hyperalgesia in regions innervated by the second branch of the trigeminal nerve (Okada-Ogawa et al., 2013). In addition, intracisternal injection of R-DIOA, an inhibitor of KCC2, induces hyperalgesia in naïve rats (Okada-Ogawa et al., 2013). Na^+^-K^+^-Cl^–^ cotransporter 1 (NKCC1), known as another regulator of intracellular Cl^–^, does not influence its expression in the medullary dorsal horn after formalin injection in the vibrissa pad (Wu et al., 2009). Thus, besides regulating the excitatory transmission, BDNF also can affect the GABAergic effect by reducing the inhibition or even reversing to excitation. The reports that pharmacological inhibition of α6GABAA receptors, a subunit of GABA receptor, reduced the enhanced pain threshold in chronic constriction injury (CCI) model rats strongly suggest that GABAergic disinhibition plays an important role in neuropathic pain. BDNF secreted from microglia can phosphorylate GluN2B via Fyn kinase, downstream signaling of TrkB (Hildebrand et al., 2016). Furthermore, it activates NMDA receptors in presynaptic terminals of primary afferent fibers, facilitating glutamate release (Chen et al., 2014). More recently, BDNF from microglia has been shown to cause an increase in the number of synaptic terminals in CGRP positive primary sensory fibers, leading to long-term potentiation in the spinal cord during neuropathic pain (Zhou et al., 2019). Thus, BDNF can regulate both excitatory and inhibitory neurotransmission. The activation of microglia is not restricted to episodes of trigeminal nerve injury. Microglia release cathepsin S, a lysosomal cysteine protease, which induces fractalkine expression on the membrane surface of neurons. Fractalkine binds to CX3CR1, a receptor exclusively expressed in microglia, and sustains their activation state (Clark et al., 2007). Prolonged release of proinflammatory cytokines from microglia leads to severe pain. It is widely accepted that microglia are involved in the development of neuropathic pain. However, minocycline, an inhibitor of microglial activation, has little effect on existing pain. This might be due to the phase shift of glial activation after nerve injury. Prominent astrocytic activation can be seen after microglial activation. In fact, intracisternal injection of fluoroacetate, an inhibitor of reactive astrocytes, attenuates neuropathic pain caused by inferior alveolar nerve injury (Okada-Ogawa et al., 2009). Physiologically, astrocytes are known to modulate neuronal function as follows: glutamate, an essential factor in excitatory neurotransmission, is synthesized from glutamine derived from astrocytes in the presynaptic terminal. Adjacent astrocytes in the gap junction synchronize their activity as neuronal assemblies (Allen and Eroglu, 2017). Suppression of excessive astrocyte activity by methionine sulfoximine, an inhibitor of glutamine synthetase, or carbenoxolone, a gap junction blocker, significantly attenuates nociceptive behavior caused by pulpitis, or infraorbital nerve transection, respectively (Tsuboi et al., 2011). The involvement of astrocyte-derived D-serine in orofacial pain is as follows: D-serine works as a co-agonist of NMDA receptors, facilitating C-fiber-mediated long-term potentiation (Kronschlager et al., 2016). D-amino acid oxidase attenuates neuropathic pain caused by infraorbital nerve injury (Dieb and Hafidi, 2013). Together, abnormal regulation of neuronal function by glial cells contributes to the induction and maintenance of orofacial pain (Figure 2).

After trigeminal nerve injuries, such as the inferior alveolar nerve, mental nerve, and infraorbital nerve transection, the primary afferent neurons become hyperexcitable, resulting in satellite glial cell activation within TG. After hyperactivation of Vc and C1-C2 nociceptive neurons, microglial cells and astrocytes are further activated (Iwata and Sessle, 2019). As mentioned earlier, minocycline is known to inhibit the activation of microglia, and bioactive substances and other cytokines, such as nitric oxide, prostaglandin, IL-1, IL-1β, IL-6, are involved in the modulation of microglial activities in the central nervous system (Iwata and Sessle, 2019). A recent study demonstrated that minocycline administration relieves orofacial neuropathic pain and partially recovers cortical activity changes induced by the partial ligation of the infraorbital nerve (Zama et al., 2019).

Therefore, in the near future, minocycline may be used as a therapeutic agent for orofacial neuropathic pain. Additionally, clinical research should focus on developing drugs for orofacial neuropathic pain with relatively few side effects.

## Diagnosis of Orofacial Neuropathic Pain

Recently, the International Classification of orofacial pain (ICOP) was described that orofacial neuropathic pain as “Orofacial pain attributed to lesion or disease of the cranial nerves” (ICOP, 2020). Neuropathic pain in the orofacial area has unique problems compared to neuropathic pain in the spinal cord. The international headache society reported that anatomical boundaries and associated medical specialty demarcations in the orofacial area contributed to the problem (ICOP, 2020). For example, the symptoms of neuropathic pain in the orofacial area often mimic odontogenic toothache, and it can easily be misdiagnosed and misdirected treatment (Christoforou et al., 2015; Christoforou, 2018). The orofacial area consists of many kinds of structures innervated by the trigeminal system, head, sinus, masticatory musculature, temporomandibular joint, jaw, teeth, and gingiva, and the complexity induces many kinds of non-odontogenic toothache (Schames et al., 2016). Besides, the measurement of orofacial pain is difficult because of its subjective nature. Therefore, accurate diagnosis of the cause of the pain is critical to avoid unnecessary dental treatment.

Orofacial neuropathic pain is thought to be classified into two types: episodic or continuous. The typical example of episodic neuropathic pain is trigeminal neuralgia (TN). TN involving the mandibular nerve, the third branch of the trigeminal nerve, is the most common type of TN, and paroxysmal pain is often felt in the tooth and can lead to the diagnosis of endodontic pain and unnecessary endodontic treatment (Antonaci et al., 2020). Diagnosis of TN is based on the patient’s history and description of the symptoms. Therefore, a detailed interview with the patient is essential to obtain an accurate diagnosis. The pain of TN is characterized by sudden and severe attacks of electric shock-like shooting pain, lasting from a few seconds up to 2 min. These can be precipitated by mild stimulation of the orofacial region from eating, brushing the teeth, speech, or putting on makeup. Classical TN is associated with chronic vascular compression of the trigeminal nerve at the root entry zone of the brainstem. Other morphological changes, such as multiple sclerosis and space-occupying lesions, can cause secondary TN (ICOP, 2020). Therefore, to diagnose TN, investigations, as well as detailed interviews, are necessary. These include cranial nerve examination, magnetic resonance imaging (MRI), and magnetic resonance angiography (MRA) of the brain.

The typical example of continuous neuropathic pain is post-traumatic trigeminal neuropathic pain, although some cases may be episodic, from minutes to days. The orofacial area may be caused by third molar extractions, implants, root canal therapy, orthognathic surgery, or facial fractures. The typical description of pain is continuous burning and/or shooting pain in the area of injury or in the distal dermatome of the affected nerve. In its early stages, intraoral post-traumatic trigeminal neuropathic pain is also often misdiagnosed as odontogenic pain (Baad-Hansen and Benoliel, 2017). Therefore, the detailed history of trauma and the classical signs of post-traumatic trigeminal neuropathic pain lead to an accurate diagnosis as well. Therefore, in order to diagnose post-traumatic trigeminal neuropathic pain, it is essential to know the patient’s specific medical history and to perform chairside sensory testing, including quantitative sensory testing (QST) (Devine et al., 2018; Jaaskelainen, 2019). Post-traumatic trigeminal neuropathic pain is defined as a “unilateral or bilateral facial or oral pain following and caused by trauma to the trigeminal nerve (s), with other symptoms and/or clinical signs of trigeminal nerve dysfunction, and persisting or recurring for more than 3 months” (ICOP, 2020). Trigeminal postherpetic neuralgia is another typical example of continuous neuropathic pain. It is a complication of herpes zoster (HZ), and occurred following trigeminal neuropathic pain attributed to herpes zoster, that is also easily mistaken for toothache and induces unnecessary dental treatment in the early stage (Paquin et al., 2017). Although the lesions heal within a few months, the infection by HZ induces painful trigeminal neuropathy. Overall, approximately 10–15% of HZ patients will develop trigeminal postherpetic neuralgia. In patients older than 60 years, trigeminal postherpetic neuralgia occurs in more than 50% (Bayat et al., 2015). It has similar clinical features to post-traumatic trigeminal neuropathic pain: allodynia and hyperalgesia to mechanical and thermal stimuli, and burning, shooting, or electric shock-like pain. Accordingly, details of the patient’s history, and sensory testing are also necessary for the diagnosis of trigeminal postherpetic neuralgia (ICOP, 2020).

There are many other neuropathic pains such as painful polyneuropathy, post-stroke pain, peripheral neuritis, glossopharyngeal neuralgia, occipital peripheral neuralgia, superior laryngeal neuralgia, and nervous intermedius neuralgia. These latter neuropathic pains are not common but need an appropriate strategy for diagnosis.

Neuropathic pain induces ectopic or extraterritorial pain upon nerve injury in the spinal area and orofacial area. However, in the orofacial area, the referred pain can easily lead to misdiagnosis as dental pain and unnecessary and irreversible dental treatment such as pulpectomy or tooth extraction. The referred pain has been explained by neuronal convergence and sensitization theories (Woolf, 2011; Markman, 2014), but it is difficult to clarify all the phenomena by referred pain. Recently, the mechanism of referred pain has been explained by various molecular and cellular changes in peripheral and central neuronal and non-neuronal cells, as above stated. The use of inhibitors and modulators for these cells in clinical trials has shown some encouraging results but still inconsistent levels of efficacy (Sainoh et al., 2016; Roerink et al., 2017). Further developments of these medications might make management of orofacial neuropathic pain and avoidance of mistreatment much easier for the clinician in orofacial neuropathic pain.

## Clinical Treatment of Orofacial Neuropathic Pain

In comparison with other neuropathic pain conditions such as post-herpetic neuralgia, painful diabetic neuropathy, and painful spinal traumatic neuropathy with a drug response rate of 20–40% (Bates et al., 2019), the response rate of PTTN is reported to be low, at approximately 11% (Haviv et al., 2014). However, the most common orofacial neuropathic pain syndromes are PPTTN as well as trigeminal neuralgia and PHTN. PPTTN is caused by trigeminal nerve injury, following procedures such as pulp extirpation, apicectomy, tooth extraction, or routine endodontic treatment. It is estimated that 3–5% of all such treatments lead to PPTTN (Baad-Hansen and Benoliel, 2017). PPTTN typically causes unilateral, continuous burning pain, at the site of injury or distal to the site, possibly with an additional sharp, shooting quality (Okada-Ogawa et al., 2015). Sensory loss may also be present. The peripheral nervous system seems to be involved at first, with additional CNS involvement over time. To manage this condition, it may be desirable to administer topical and/or systemic therapy to target the peripheral or central nervous system or both.

## Topical Therapy

The topical application of medication is a relatively new method to treat neuropathic pain. Local drug delivery has advantages over centrally acting drugs that must taper up to adequate levels: it is less likely to induce systemic side effects or interact with other medications and can provide faster relief (Nasri-Heir et al., 2013). Discontinuation of centrally acting medications can also cause side effects, a phenomenon far less common with topically applied medications. Abrupt discontinuation of systemic medication can sometimes be dangerous, occurring when refills are not done on time or when patient instructions are ignored, misunderstood, forgotten, or not given (Sheikh et al., 2013). Topical medications are usually used to treat a specific peripheral target, making them useful for PPTTN, which involves peripheral nerve sensitization (Padilla et al., 2000). For severe pain, a combination of systemic and topical medications is often required. Topical lidocaine can desensitize a painful site by blocking a peripheral ectopic generator, such as a triggering zone near the tissue surface, in trigeminal neuralgia (Zakrzewska, 2010). It has also been shown to provide pain relief for PHTN patients (Derry et al., 2014). Five-percent lidocaine patches offer excellent comfort from allodynia in patients with slight sensory loss, but not in those with profound sensory loss.

With only preliminary evidence of benefit, another topical medication is orally applied capsaicin (Zostrix 0.025 or 0.075%) recommended for patients with orofacial neuropathic pain (Padilla et al., 2000). Initially, most patients complain of a burning sensation that wears off within 1–2 days. Repeated capsaicin applications alleviate pain by depleting SP in C-fiber primary afferents, thereby reducing the peripheral noxious inputs. A combination of capsaicin and Orabase (a paste containing gelatin 16.7%, pectin 16.7%, carboxymethylcellulose Sodium 16.7%) can be applied topically to the injured area using a neurosensory stent (Padilla et al., 2000). If sympathetic involvement is suspected, clonidine, an alpha-2-adrenergic agonist, can be added to the formulation (Epstein et al., 1997). This drug causes the suppression of the norepinephrine release from sympathetic terminals. Some clinicians have reported that a topically-applied formulation containing amitriptyline 2% and ketamine 1% has an analgesic effect against orofacial neuropathic pain (Sawynok and Zinger, 2016).

## Systemic Pharmacotherapy

International and regional professional associations have published clinical practice guidelines on the pharmacological management of neuropathic pain. The most commonly recommended, centrally targeted analgesics are tricyclic antidepressants (TCAs; e.g., amitriptyline and nortriptyline), serotonin-norepinephrine reuptake inhibitors (SNRIs; e.g., venlafaxine and duloxetine), gabapentin and pregabalin, the latter two being the first drugs used for PPTTN (Lewis et al., 2007). The combination therapy of duloxetine or amitriptyline plus gabapentin or pregabalin should be the second choice for PPTTN. If the above strategy fails, opioids and opioid combinations may be a viable alternative. Pregabalin and gabapentin are classified as anticonvulsants. These two drugs inhibit the release of excitatory neurotransmitters such as glutamate and SP by combining with the α2δ subunit of the voltage-gated calcium channel in the central nervous system. Clinically, pregabalin and gabapentin are useful for persistent pain and numbness associated with PHTN, and diabetic neuropathy (Snedecor et al., 2014). Large clinical trials of pregabalin and gabapentin on PHTN patients demonstrated significant effects on PHTN pain; this analgesic effect is similar to that found in studies involving antidepressants. Mirogabalin is a newly synthesized, potent, and selective α2δ ligand, another gabapentinoid. It has been approved in Japan for the treatment of peripheral neuropathic pain, including painful diabetic peripheral neuropathy and PHTN (Kato et al., 2019). An animal study demonstrated that mirogabalin alleviated tactile allodynia in CCI of the sciatic nerve in rats (Murasawa et al., 2020). The effects of mirogabalin may be partly mediated by reducing ectopic afferent activity, thus directly reducing or eliminating the nociceptive afferent input to the medullary dorsal horn. A recent study demonstrated that the pain-inhibitory system became less sensitive to drugs in PPTTN patients over time (Nasri-Heir et al., 2015). Therefore, impaired inhibitory pain modulation should be considered a target in the management of PPTTN patients. TCAs or SNRIs are thought to block the reuptake of norepinephrine and serotonin (Nasri-Heir et al., 2015). Some clinicians have reported that among the various analgesic drugs available for treating neuropathic pain, TCAs provide the greatest benefit for patients with PPTTN (Nasri-Heir et al., 2015). SNRIs, such as duloxetine and venlafaxine, have also been used for orofacial neuropathic pain management. The central pain modulation, aided by TCAs or SNRIs, originates from brainstem neurons, mediated by the noradrenergic or serotonergic systems, and results in the enhancement of the pain inhibitory system.

On the contrary, carbamazepine, and oxcarbazepine, the first-line recommended medications in a systematic review, reduce pain in approximately 90% of patients with trigeminal neuralgia. Baclofen, lamotrigine, pregabalin, and gabapentin are included in the treatment for classical trigeminal neuralgia as a second-line treatment (Moore et al., 2019). To improve the effectiveness of oral anticonvulsants and abort an acute attack, the use of topical, injected, or intravenous lidocaine, intravenous fosphenytoin, and topical or injected sumatriptan are recommended in the current practice (Moore et al., 2019).

Pre-emptive analgesia is the possible treatment wherein preoperative treatment is designed to prevent the development of orofacial neuropathic pain. This treatment aims primarily to avoid the initial injury-induced afferent volley and central sensitization by using local anesthetic blocks during invasive dental procedures or oral surgery and diminishing the production of inflammatory mediators. Preventive analgesia focuses on the relative timing of preemptive analgesia or anesthetic interventions. It attenuates the impact of the peripheral nociceptive transduction associated with noxious stimuli preoperatively, intraoperatively, and/or postoperatively (Korczeniewska et al., 2020). In the dental setting, clinicians should consider using local analgesia during invasive dental procedures or using preemptive analgesics and/or anti-inflammatory drugs to prevent postoperative pain.

## Conclusion

Following trigeminal nerve injury, non-neuronal cells, astrocytes, microglial cells, and astroglial cells, are activated, and macrophages are accumulated. The various molecules are generated in these non-neuronal cells and released from them, and these molecules are involved in the enhancement of the noxious neuronal activity. The glial cell-neuron and glial cell-glial cell interactions in TG, Vc, and C1/C2 regions are crucial mechanisms underlying orofacial neuropathic pain. According to the recent findings obtained from basic animal studies, appropriate diagnostic parameters and a variety of new treatments were developed for orofacial neuropathic pain and trigeminal neuropathy patients. More detailed mechanisms of non-neuronal cell function need to be evaluated in future studies, and the basic data hope to be applied for the appropriate diagnosis and treatment of orofacial neuropathic pain patients.

## Author Contributions

MS, YH, SH, and KI contributed to conception, design, analysis, interpretation, drafted and critically revised the manuscript. YI contributed to the concept, design, analysis, interpretation, drafted and critically revised the manuscript. NN and AO-O contributed to conception, analysis, interpretation, drafted about clinical treatment of orofacial neuropathic pain or diagnosis of orofacial neuropathic pain, respectively, and critically revised the manuscript. All authors gave their final approval and agreed to be accountable for all aspects of the work.

## Conflict of Interest

The authors declare that the research was conducted in the absence of any commercial or financial relationships that could be construed as a potential conflict of interest.

## Abbreviations

AMPA: alpha amino-3-hydroxy-5-methyl-4-isoxazole-propionate
BDNF: brain-derived neurotrophic factor
CCI: Chronic Constriction Injury
CCL2: the chemokine C-C motif ligand 2
C1-C2: the upper cervical spinal cord
Cx43: Connexin 43
HZ: herpes zoster
IASP: International Association for the Study of Pain
IGF-1: insulin-like growth factor-1
IL: interleukin
KCC2: K^+^-Cl^–^ cotransporter
MCP-1: monocyte chemoattractant protein-1
MRA: magnetic resonance angiography
MRI: magnetic resonance imaging
MT: medial thalamic nuclei
Nav1.8: voltage-gated sodium channels 1.8
NMDA: *N*-methyl -D-aspartate
PBN: parabrachial nucleus
QST: quantitative sensory testing
sEPSCs: spontaneous excitatory postsynaptic currents
sIPSCs: spontaneous inhibitory postsynaptic currents
SNRIs: serotonin-norepinephrine reuptake inhibitors
SP: Substance P
PHTN: Postherpetic trigeminal neuralgia
PPTTN: peripheral, painful, traumatic trigeminal neuropathy
TCAs: tricyclic antidepressants
TG: trigeminal ganglion
TN: trigeminal neuralgia
TNFR: TNF receptor
TNFα: tumor necrosis factor alpha
TrkB: tropomyosin-related kinase B
Vc: trigeminal spinal subnucleus caudalis.
